# Attention allocation and social worries predict interpretations of peer-related social cues in adolescents

**DOI:** 10.1016/j.dcn.2017.03.004

**Published:** 2017-03-18

**Authors:** Simone P.W. Haller, Brianna R. Doherty, Mihaela Duta, Kathrin Cohen Kadosh, Jennifer Y.F. Lau, Gaia Scerif

**Affiliations:** a Department of Experimental Psychology, University of Oxford, Oxford, United Kingdom; b Institute of Psychiatry, Psychology & Neuroscience, King’s College London, London, United Kingdom; c School of Psychology, University of Surrey, Surrey, United Kingdom

**Keywords:** Adolescence, Social anxiety, Eye tracking, Attention, Appraisal, Interpretation

## Abstract

Adolescence is a sensitive period for increases in normative but also debilitating social fears and worries. As the interpretation of interpersonal cues is pertinent to social anxiety, investigating mechanisms that may underlie biases in social cue appraisal is important.

Fifty-one adolescents from the community aged 14–19 were presented with self- and other-relevant naturalistic social scenes for 5 s and then required to rate either a negative or a positive interpretation of the scene. Eye-tracking data were collected during the free viewing period to index attentional deployment. Individual differences in social worries were measured via self-report.

Social anxiety levels significantly predicted biases in interpretation ratings across scenes. Additionally, cumulative attentional deployment to peer cues also predicted these interpretation biases: participants who spent more time on facial displays perceived more threat, i.e. endorsed more negative and less positive interpretations. Self-relevant scenes yielded greater tendencies to draw negative interpretations. Finally, older adolescents also selected more benign interpretations.

Social anxiety is associated with a bias in interpreting social cues; a cognitive bias that is also influenced by attentional deployment. This study contributes to our understanding of the possible attention mechanisms that shape cognitions relevant to social anxiety in this at-risk age group.

## Highlights

• Across scenes, increasing social anxiety was associated with greater endorsement of negative interpretations.
• Greater attentional deployment to peers predicted increased endorsements of negative interpretations.
• Self-relevant scenes yielded more negative interpretations.
• Older adolescents selected more benign interpretations.

## 1 Introduction

Social interactions are central to well-being across the life span, with different relationships (e.g., caregivers, peers, romantic relations) taking center stage at different developmental periods (Nelson et al., 2016). In adolescence, peers become increasingly important (Steinberg and Silverberg, 1986). Given increased affective and motivational value of specifically peer-related social cues during this period, it is perhaps not surprising that normative social anxiety and self-consciousness increase (Miers et al., 2014, Westenberg et al., 2004). Age-of-onset data further suggests that adolescence is a developmentally sensitive juncture for the emergence of more impairing, clinical levels of social fears and worries. These tend to persist and account for a significant proportion of adult Social Anxiety Disorder (SAD; e.g., Kessler et al., 2005).

### 1.1 Cognitive biases and social anxiety

Social interactions require the attention to and interpretation of complex and dynamic visual and verbal, often individual-specific indicators of others’ mental states. Preferential allocation of attention to socially threatening cues (e.g, faces or words) and negative interpretations of ambiguous social cues (e.g., a frown, a pause in a conversation, a smile) have been linked to social fears and worries in youths (e.g., Muris and Field, 2008). These biases are thought to shape experiences of the social world and maintain fears by increasing perceived negative social feedback (Clark and Wells, 1995; Rapee and Heimberg, 1997). Biases in the interpretation of social-evaluative situations are targeted in treatment approaches such as Cognitive Behavioural Therapy.

Surprisingly little is known about the mechanisms underlying disproportional social threat perception – how biases in one or more central cognitive processes result in a skewed representation of the social world. Biases have been suggested to permeate early to late stages of information processing (Musa and Lépine, 2000), with interactive effects on emotional responding (Hirsch et al., 2006). It is plausible that low-level biases in attention (here operationalized as indices of attentional allocation that can be measured through eye movements) and higher-level interpretation biases (such as negative interpretations of ambiguous materials) are closely linked. They could represent a single cognitive mechanism with interpretation biases the results of downstream effects of attention biases, or negative interpretation biases habitually shaping attentional focus (Wong and Rapee, 2016, White et al., 2011). Alternatively, it may be that attention and interpretation reflect distinct cognitive mechanisms that independently link to individual differences in social anxiety. It is important to move towards integrating different stages of processing to arrive at a more comprehensive understanding of anxiety-linked information processing. Here, we assess whether naturalistic social targets that are interpreted differently are also scanned differently. We test the hypothesis that attention is a mechanism underlying the disproportional threat interpretation that is characteristic of social anxiety. An understanding of these linkages across a potential sensitive developmental period for social cognition such as adolescence may i) propel our understanding of vulnerability and risk trajectories for social anxiety in adolescents ii) help understand how changes in social cognitions and attentional control within adolescence affect clinically relevant processes.

### 1.2 Cognitive bias and social anxiety studies in developmental populations

To date, only a limited number of studies have measured interpretation bias in relation to social anxiety in adolescent samples. These studies suggest that at least by mid-adolescence, biases in the interpretation of ambiguous social-evaluative material consistently characterize adolescents with SAD and link with individual differences in social anxiety levels in adolescents from the community (Giannini and Loscalzo, 2016, Haller et al., 2016, Miers et al., 2008). It should be noted that there is some contention as to the degree to which these cognitions purely reflect a started perception of reality or are grounded to some degree in a social skill deficit (e.g., Miers et al., 2011).

Biases in *attention* allocation in adolescents with high levels of (social) anxiety have mostly been investigated in the framework of behavioral (i.e., reaction time) indices in highly controlled competitive viewing arrangements, such as the dot-probe paradigm (i.e., attention needs to be divided between two simultaneous displays, usually a valenced and a neutral cue, usually faces). Several studies have concurrently collected eye-tracking data to assess the time course of attention. Results of behavioural studies on attention biases in youth populations are relatively mixed but overall suggest a small magnitude bias towards threatening cues in higher anxious youths (Puliafico and Kendall, 2006, Bar-Haim, 2010, Dudeney et al., 2015). Those six studies that have concurrently collected eye-tracking data find equivocal results, with some studies finding differences in attention allocation between high and low anxious youths in early time windows (i.e., hyper-vigilance; Seefeldt et al., 2014, Shechner et al., 2013) and some finding evidence for differences in later time windows (i.e., avoidance; In-Albon et al., 2010, Shechner et al., 2015) and two studies finding no differences when stimuli remain on the screen for longer durations (Gamble and Rapee, 2009, Price et al., 2013). This may suggest that anxious youths are characterized by more complex attentional patterns such as hyper vigilance-avoidance, with exposure time significantly affecting temporal attention patterns. Given that the results do not consistently report biases in attention allocation when comparing displays of threatening and neutral cues, it is important to explore additional dimensions to assess how attention gates anxiety-relevant cognitions more directly.

### 1.3 Links between attention and interpretation biases

The only study that investigated the relationship between attention and interpretation within the same paradigm (in adults selected for high and low depression scores) used a simultaneous presentation of positive and negatively valenced stimuli in a scrambled sentence task (Everaert et al., 2014). The authors found that indices of selective attention (i.e., time spent on one option of sentence completion compared to another) were related to interpretations of the material. It is plausible that time delimited exposure of competing, highly valenced material drives the link between selective indices of attention and interpretation. Biases may link differently in settings where exposure times are longer and targets more ambiguous or complex.

A small body of work has further investigated causal relations between these processes by experimentally manipulating either interpretative or attentional processes. Amir et al. (2003) trained anxious individuals to endorse the benign meaning of ambiguous information. They found that individuals exhibited an improved ability to disengage attention from threatening cues post-training. Similarly, White et al. (2011) showed that inducing an attention bias in healthy volunteers using the dot-probe task affected how subsequent ambiguous information was interpreted. The authors found that increasing bias to threat resulted in increasingly negative interpretations of ambiguous material. These first studies speak to bi-directional, intricate links between interpretation and attention processes. Whether these linkages and cascading effects are also found in youth populations remains an empirical question. With developmental work pointing to prolonged maturational trajectories of attentional control and the appraisal of social cues (Kilford et al., 2016), the role of each process in anxious responding could change with development.

### 1.4 Using eye-tracking to study attentional processing in social anxiety

Eye-tracking is a useful tool to study unconstrained scanning of visual input especially when other behavioral indicators of attention allocation, such as reaction times, are not available. This is particularly useful when studying the processing of more complex, natural scenes. Studies assessing social cue processing in youths have often failed to consider the complexity of natural social interactions. The most commonly used stimuli are face stimuli, especially in the anxiety literature (e.g., Daudley et al., 2016). Across studies, selected target faces, often displayed alongside neutral faces, usually display high threat expressions that are rarely encountered in everyday life – socio-cultural conventions make it unlikely that one receives unfiltered thoughts both in terms of criticism and praise. Instead, self-relevant, negatively interpreted ambiguous or mildly threatening facial and gestural cues are likely particularly pertinent to socially anxious feelings in day-to-day experiences of youths. In order to understand how individuals understand the world differently, we arguably need to move closer to natural visual behavior that is more representative of social interactions.

Thus far, there are no studies of developmental populations that examine unconstrained scanning of in youths in relation to social anxiety. Equally, there is no work assessing the relationship between viewing and interpretations of naturalistic social scenes, neither in adults, nor developmental populations.

### 1.5 The current study

In this study, we examined scanning of social scenes, alongside interpretative processes, in adolescents from the community with varying levels of social worries. We used a modified version of the picture-based, free-viewing interpretation task (Haller et al., 2016) to measure interpretations and scanning patterns of naturalistic social scenes. We assessed the hypothesis that biases in the interpretation of ambiguous material are linked to social anxiety levels and manifest in attentional allocation while scanning visual scenes. We predicted that adolescents with increased social anxiety levels would exhibit a bias in interpreting social scenes compared to youths with low levels of social worries. We expected social anxiety levels to interact with scanning indices of attentional allocation to predict interpretations across scenes. As previous research has shown that negative interpretations are particularly pronounced when situations are processed in a self-related manner (Amin et al., 1998, Vassilopoulos and Banerjee, 2012), we further explored the effect of a self-related visual cue on viewing patterns and interpretations. As previous studies have highlighted the role of developmental status in attentional deployment to emotional stimuli (e.g., Gamble and Rapee, 2009) and interpretation bias (e.g., Creswell et al., 2014), potential age effects were also examined, although this was not the primary aim of the study. Additionally, to conform with previous publications, we analyzed pupil dilation data as a measure of cognitive and emotional processing demands (e.g., Price et al., 2013, Shechner et al., 2015, Silk et al., 2012). We report on this measure and associated methods in the Supplementary materials.

## 2 Methods

### 2.1 Sample

A total of 60 female adolescents from the community participated in the study. Only females were included in this study to reduce the variability given the wide age range. The study received ethical approval by the Central University Research Ethics Committee of the University of Oxford (CUREC). Legal guardians/parents and/or participants signed informed consent or assent prior to participation. Participants were recruited via local schools and reimbursed with a £5 Amazon gift voucher. All participants had normal or corrected-to-normal vision. Eight participants were excluded because the eye-tracking data did not satisfy the minimum number of valid trials per condition as detailed below. One participant was excluded due to failing to comply with the task instructions. The final sample consisted of 51 adolescents (age range: 14.0–19.75 years, M = 16.73, SD = 1.26, PDS Pubertal Status: mid- to late pubertal).

### 2.2 Materials and measures

#### 2.2.1 Eye-tracking apparatus

Eye movements and pupil dilation were recorded using a Tobii TX300 eye-tracker, collecting binocular data at 300 Hz. All calibration and task stimuli were presented using custom routines implemented in MATLAB 2012a (The MathWorks Inc., MA) using Psychophysics Toolbox (Brainard, 1997).

#### 2.2.2 Stimuli

**Fig. 1:**
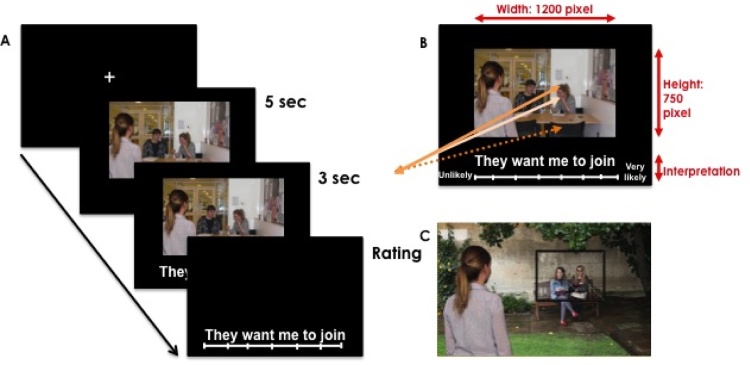
Stimulus presentation, trial structure and Areas of Interest (AOIs). A. Trial sequence B. Example stimulus and screen division C. AOI. Black square marks peer AOI.

The stimuli were presented on a 24-inch monitor (1920 × 1080 pixels; 94ppi, 60 Hz) situated 57 cm away from participants (51.7° by 29.1° visual angle). Stimuli were 72 colour photographs of complex social scenes (1200 by 750 pixels), spanning 32.31° by 20.12° of visual angle. The scenes were presented against a black background off center vertically at 2.65° visual angle (see Fig. 1B).

Each scene was associated with two interpretation statements and a visual analogue scale (VAS). There were two types of interpretation statements (i) a statement with a positive valence (e.g., *They want me to join them for break; They want to tell me about the annoying new teacher*) or (ii) a statement with a negative valence (e.g., *They do not want me to join them; They don’t want me to study with them*).

The scenes included a Protagonist seen from the back in interaction with peers. Peer portrayals ranged from positive to negative expressions and gestures. In a previous study using a subset of the scenes, we showed that scenes varied on a continuum of ambiguity with even the most valenced scenes appraised differently across participants (Haller et al., 2016). To ensure that scenes assigned to Self and Other condition were matched as closely as possible, we coded them for positive and negative gestures. Scene assignment to Self and Other conditions was done by matching on gesture valence, number and gender of peers portrayed in the scene.

The Protagonist was either a picture of the participant (Self trial) or a picture of a gender-matched female (Other trial) inserted on the right or left side of the scene. Two distinct ‘Other’ Protagonists (light hair, dark hair) were chosen to mismatch participants’ own hairstyles to ensure a pronounced difference between the self-other trials. Sets were counterbalanced between participants with each scene only appearing once for each participant, either in the Self or Other condition with either a positive or negative interpretation statement.

#### 2.2.3 The interpretation task

After participants looked continuously at the screen for at least 200 milliseconds (ms), the trial started with a fixation cross with a duration jittered between 1–2 s (s). The fixation was followed by the presentation of the scene on its own for 5 s, followed by the presentation of the scene accompanied by an interpretation statement (either positive or negative) for a further 3 s, which was in turn followed by the presentation of the interpretation statement on its own accompanied by a visual analogue scale (VAS) underneath. The scene remained on the screen during the first presentation of the interpretation statement to encourage an online interpretation and avoid a memory component to the interpretation rating. Participants used the mouse to choose a point on the scale that reflected how likely it was that the Protagonist understood the situation in the way the statement described it. The trial ended as soon as the participant completed the rating. The inter-trial interval was jittered between 5–6 s. See Fig. 1A for a visual representation of the trial sequence.

The order of the scenes was randomized across and within 6 blocks of 12 scenes. An initial practice trial allowed participants to familiarize themselves with the trial sequence and their own photograph.

#### 2.2.4 Measures

##### 2.2.4.1 Social anxiety measure

Social anxiety was assessed using the Social Anxiety Scale for Adolescents (SAS-A; La Greca and Lopez, 1998). The SAS-A is a 22-item self-report measure including 18 statements pertaining to social anxiety (e.g., *I feel shy around people I don’t know*) and four filler items (e.g., *I like to read*). Previous studies have reported good internal consistency and adequate test-retest reliability (e.g., Storch et al., 2004). Recommended cut-off scores for clinically significant levels of social anxiety have been suggested between 50 and 54 (e.g., Tulbure et al., 2012, La Greca and Lopez, 1998). The mean social anxiety score across the whole sample was M = 46.90, SD = 10.60, range = 28-74, which is comparable with levels reported in previous studies and normative data for females (La Greca and Lopez, 1998).

##### 2.2.4.2 Puberty measure

The Peterson Development Scale (PDS; Petersen et al., 1988) is a self-report, non-intrusive measure of pubertal status. Reliability estimates for the PDS are reported in the “good” range (Petersen et al., 1988). Questions pertained to 1) occurrence of a growth spurt, 2) changes in complexion, 3) the development of body hair 4) breast development and 5) the onset of menarche. Participants rated their development using a four-point scale, ranging from “…not yet begun” = 1 to “…seems completed” = 4, with the exception of the menarche question (“Yes” = 4 points or “No” = 1 point). The total sum score of the first five questions was divided by 5 for the category metric reported in the sample description.

### 2.3 Procedure

Participants were tested individually in a soundproof, dimly lit room at the Department of Experimental Psychology. Prior to the experimental session, a single photograph of each participant was taken from the back and superimposed onto the set of experimental stimuli using MATLAB 2012a (The MathWorks Inc., MA) and a graphics software suite (Gimp, Version 2.6). Participants first completed the self-report questionnaires. Participants were then seated in front of the eye-tracker and a 9-point calibration (20%, 50% 80% of both horizontal and vertical display span) was run. Calibration was considered satisfactory if at least 12 gaze samples within one degree of visual angle were collected per calibration point in the screen area corresponding to stimuli presentation. The task started with instructions displayed on the screen and followed by a practice trial that exposed the participant to her own photograph for the first time. The experimenter assisted the participant during the practice to make sure that she understood the instructions. The task was approximately 25 min long and was split into three parts separated by short breaks. At the end of the experimental session, participants were debriefed about the nature of the research and received the gift voucher.

### 2.4 Data processing

#### 2.4.1 Cleaning and artifact rejection

Eye-tracking data was processed using custom MATLAB routines. Gaze data was filtered with a second-order Savitsky-Golay filter with a length of 7 samples (23 ms) (Nyström and Holmqvist, 2010). Pupil diameter data was smoothed with a 3-sample (100 ms) median filter. Pupil diameter was baseline corrected with respect to the mean of the 200 ms prior to scene onset. Eye-tracking data was considered valid if (i) the eye-tracker validation flag indicated that both eyes were found, (ii) the recorded gaze was within the screen area, and (iii) the recorded pupil diameter was positive and within physiological range. Blinks were detected as sections of the data with instantaneous rate of change of pupil diameter greater than 0.1 mm for both eyes and the corresponding samples were flagged as invalid for both gaze and pupil data. The invalid data for gaze was replaced with last valid value, while the invalid data for pupil diameter was linearly interpolated. Trials were excluded from analysis if they had i) more than 1000 ms consecutive invalid points after fixation onset, ii) more than 1000 ms consecutive invalid points prior to scene offset or iii) more than 40% of invalid points between fixation onset and scene offset. Participants were excluded from the analysis if they had less than 30 valid trials per condition. Drift was corrected per trial with respect to the fixation on the initial cross. This drift correction was applied only if it required less than 150 pixels, i.e., 3.5°. An overall y gaze correction with respect to the initial fixation cross was also applied for four participants. Analysis of gaze and pupil was based on the left eye data only during the presentation of the scene on its own.

#### 2.4.2 Gaze data processing

Fixations were determined based on a maximum gaze velocity threshold of 75° visual angle/second, a dispersion threshold of 2° visual angle around the fixation centroid and a minimum duration threshold of 75 ms. Areas of interest (AOIs) were defined for each scene to include the face region of the peers portrayed in the picture (see Fig. 1C). Total fixation time on the AOIs during the 5000 ms presentation window were calculated.

#### 2.4.3 Statistical plan

Gaze data (i.e., total fixation times) for each AOI during Self and Other trials were extracted for the 5 s free viewing period during the presentation of the scene alone. Interpretation ratings of the VAS scale were extracted as response percentage out of 100 with ‘0′ representing the left end of the scale (Unlikely) and ‘100′ representing the right of the scale (Very Likely) for positive and negative interpretation statements respectively.

We used linear mixed effects models to examine whether gaze and individual differences measures (social anxiety, age) predicted positive and negative interpretation ratings across scenes using the *lme4* package (Bates et al., 2015) in *R*. To establish the significance of effects, an information-theoretic (IT) approach using Akaike’s information criterion (AIC) modelling (Burnham and Anderson, 2002) was used. In this approach, a global linear mixed-effects model was created using all fixed predictor variables of interest, with subject and scene as random variables to account for the non-independence across trials within participants and across participants within scenes. Next, a subset of candidate models that contained all possible combinations of the fixed effects included in the global model were specified. Akaike weight-based averaging over all candidate models allowed for determining the mean estimates of the coefficients (*θ*) weighted by the Akaike weight (*w*) as well as the 95% confidence intervals (CI) used to determine which coefficients were statistically significantly different from zero. This model averaging was performed using the *R* package *MuMIn* (Bartón, 2015).

Two models were run to determine if gaze measures as well as age and social anxiety predicted interpretation ratings. The two models included: 1) perspective (self, other), interpretation statement valence (positive, negative), fixation time, and social anxiety as well as all interactions among variables, and 2) perspective (self, other), interpretation statement valence (positive, negative), fixation time, and age as well as all interactions among variables.

## 3 Results

For the first model, the coefficient estimate for social anxiety was significantly different from zero as well as the coefficient estimates for the two-way interactions between statement valence and perspective, statement valence and fixation time, and statement valence and social anxiety (see Table 1 for the full model). There were no additional significant terms.

**Table 1:** Full model examining Social Anxiety, Perspective, Fixation Time and Statement Ratings.

Fixed effect	Estimate	95% CI	*p*-value
**(Intercept)**	**50.81**	**[48.56, 53.06]**	**<0.001**
Perspective	−1.46	[−4.49, 1.55]	0.346
Statement valence	−2.89	[−5.95, 0.16]	0.064
Fixation time	1.85	[−0.20, 3.88]	0.076
**Social anxiety**	**2.87**	****[1.12, 4.60]****	**0.001**
Perspective*Statement valence	**4.16**	****[0.24, 8.08]****	**0.038**
Perspective*Fixation time	2.69	[−0.06, 5.46]	0.056
Statement valence*Fixation time	**−4.82**	****[−7.38, −2.26]****	**<0.001**
Statement valence*Social anxiety	**−4.52**	****[−6.57, −2.43]****	**<0.001**
Perspective*Statement valence*Fixation time	−2.92	[−6.87, 1.04]	0.148
Perspective*Social anxiety	−0.78	[−3.00, 1.48]	0.503
Fixation time*Social anxiety	0.07	[−1.06, 1.25]	0.911
Perspective*Statement valence*Social anxiety	1.25	[−2.67, 5.16]	0.533
Statement valence*Fixation time*Social anxiety	0.27	[−1.71, 2.27]	0.794
Perspective*Fixation time*Social anxiety	0.80	[−1.19, 2.80]	0.434
Perspective*Statement valence*Fixation time*Social anxiety	0.20	[−3.79, 4.19]	0.922

**Fig. 2:**
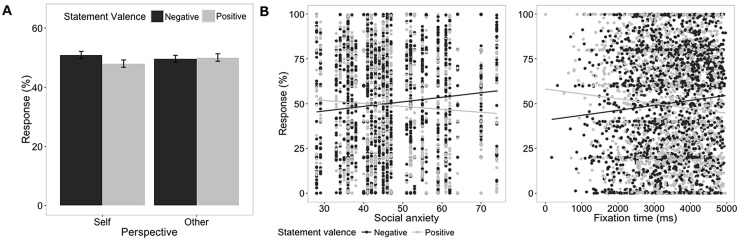
Interactions between variables of Model 1. A. Self-related scenes received increased negative and decreased positive interpretation ratings. B. Individual differences in social worries predicted negative and positive interpretation ratings. Fixation time also predicted interpretation ratings.

The interaction between statement valence and social anxiety showed a positive relationship between social anxiety and interpretation ratings for negative interpretations and a negative relationship for positive interpretations. Participants higher in social anxiety were more likely to interpret scenes more negatively and less positively. The interaction between statement valence and perspective showed increased negative and decreased positive statement ratings for self-relevant scenes, but no difference between positive and negative interpretations for other-relevant scenes. Hence, adolescents evaluated social situations as more negative and less positive if these were self-relevant. The interaction between statement valence and fixation time showed a positive relationship between fixation time and response percentage for negative interpretations and a negative relationship for positive interpretations. Hence, participants who spent less overall time on peer AOIs rated positive interpretations higher and negative interpretations lower (see Fig. 2 for a visual illustration of all interactions).

**Fig. 3:**
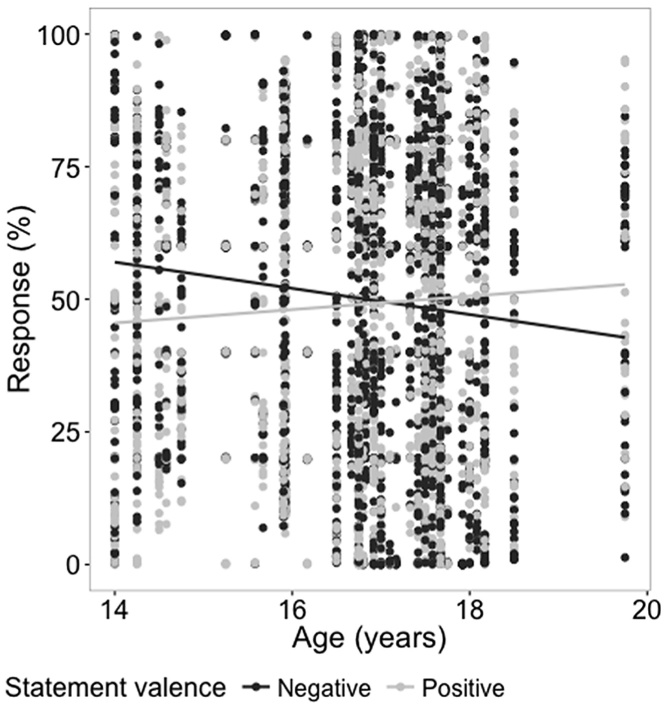
Interaction between age and statement valence. For analyses of the pupil data please see Supplementary materials.

For the second model, the same two-way interactions between statement valence and perspective, and statement valence and fixation time emerged (see Table 2 for the full model). Additionally, coefficient estimates of age were significantly different from zero. An additional significant two-way interaction between statement valence and age emerged. The interaction between statement valence and age showed a positive relationship between age and interpretation rating for positive interpretation statements and a negative relationship with negative interpretation statements. With increasing age, participants were more likely to endorse positive and less likely to endorse negative interpretations (Fig. 3).

**Table 2:** Full model examining Age, Perspective, Fixation Time and Statement Ratings.

Fixed effect	Estimate	95% CI	*p*-value
**(Intercept)**	**50.75**	**[48.51, 53.03]**	**<0.001**
Perspective	−1.49	[−4.54, 1.46]	0.335
Statement valence	−2.87	[−5.95, 0.13]	0.067
**Age**	**−3.58**	**[−5.33, −1.81]**	**<0.001**
**Fixation time**	**2.34**	****[0.30, 4.33]****	**0.024**
Perspective*Statement valence	**4.22**	**[0.30, 8.14]**	**0.035**
Perspective*Fixation time	2.58	[−0.14, 5.30]	0.063
Statement valence*Age	**5.44**	**[3.34, 7.53]**	**<0.001**
Statement valence*Fixation time	**−5.58**	****[−8.11, −3.02]****	**<0.001**
Perspective*Statement valence*Fixation time	−2.79	[−6.74, 1.16]	0.167
Age*Fixation time	1.00	[−0.44, 2.43]	0.173
Statement valence*Age*Fixation time	−1.16	[−2.99, 0.68]	0.218
Perspective*Age	0.49	[−1.78, 2.70]	0.675
Perspective*Age*Fixation time	−1.49	[−3.32, 0.36]	0.118
Perspective*Statement valence*Age	−0.88	[−4.88, 3.11]	0.668
Perspective*Statement valence*Age*Fixation time	1.08	[−2.61, 4.77]	0.567

## 4 Discussion

The objective of the current study was to assess whether individual differences in social worries interact with attentional scanning to predict interpretations across social scenes. We used a picture-based interpretation task that required participants to evaluate positive and negative interpretations to complex social scenes after a 5 s free-viewing period.

We did not find that social anxiety interacted with fixation times to predict interpretations. However, we found that individual differences in social worries and total fixation time independently predicted positive and negative interpretation ratings. Individual differences in social anxiety predicted interpretation ratings such that adolescents from the community with higher social anxiety levels rated negative interpretations as more likely and positive interpretations as less likely across both self- and other-related scenes. Additionally, fixation times predicted interpretations across scenes: adolescents who spent overall more time on the peer AOIs perceived more threat, i.e., rated negative interpretations as more likely and positive interpretations as less likely. Additionally, we found that across participants social situations that contained a self-referential cue (i.e., one’s own photograph) were rated more negatively and less positively than scenes rated for an unknown other. Further, age also played a role in interpretation ratings such that younger (i.e., mid-adolescent) youths rated interpretations less benign across scenes: mid-adolescent youths rated negative interpretations as more likely and positive interpretations as less likely. We discuss each of these results in turn.

The results on the association between attentional allocation and interpretation ratings provide evidence that, even when probed with centrally presented single complex displays with long exposure times (in comparison to competing displays of multiple stimuli at short duration times), attentional allocation and interpretation are closely interlinked processes. The directionality of findings (i.e., longer total fixation time on peer facial AOIs linked to increased threat interpretation) is particularly interesting in the light of a recent study by Shechner et al. (2015). The authors found that the magnitude of attentional avoidance in a competitive viewing arrangement was correlated with post-task self-reported aversive intensity ratings of the scenes (although this was only the case in adults, in adolescents no association was found between attentional avoidance and post-task picture ratings). Hence, attention allocation may link differently to interpretation depending on the context (e.g., singular face targets, competing face displays, naturalistic scenes). Singular social targets embedded in complex naturalistic scenes may hold attention as a function of increased threat perception in adolescents.

We did not find that individual differences in social worries interacted with self-relevance or attention to predict interpretation ratings. There is some evidence that biases in interpretations are more pronounced when the social material is self-referential (e.g., Amin et al., 1998, Vassilopoulos and Banerjee, 2012). Previous studies required participants to rate several interpretations for each scene (Miers et al., 2008, Haller et al., 2016). We only displayed a single interpretation for each scene; while we did find associations between social anxiety levels and absolute interpretation ratings across scenes, evaluating two interpretations for each scene may result in stronger links between ratings and social anxiety and also bring out effects of self-relevance that may be subtler.

It may be that social anxiety by attention interactions would emerge in a selected or clinical sample, especially given that social anxiety severity has been shown to moderate vigilance to threat cues (Bantin et al., 2016). It is also plausible that interactions are more likely to emerge in competitive viewing arrangements, when attention needs to be shifted and is divided between several, in-congruent cues. In comparison to previous, highly controlled studies, the interpretation task used in this study required participants to deploy attention in a goal-directed manner. The central peer-related cues were relevant to the interpretative task that participants were asked to perform, hence, assessing social-evaluative ambiguity was related to the goal that is pursued (interpretation). The explicit interpretation task likely resulted in increased processing of social-evaluative information for all participants and may therefore obscure, as opposed to bring out, social anxiety-related differences in non-goal driven attentional capture. Probing motivational and functional aspects of attention is important as attention is deployed in a goal-directed manner in every day life (Allport, 1989, Norman and Shallice, 1986).

It is also possible that normative developmental effects of adolescence in general, such as increased interest in social cues (and therefore age-related increases in attentional deployment to social cues) and elevated social concerns, may ‘wash out’ differences specifically in this age group in a non-clinical sample. Shechner et al. (2015) found that adolescents aged 8–17 overall spent more time on socially threatening stimuli than adults when simultaneously presented with neutral and non-social threat. Developmental data is needed to understand the degree to which age effects, especially adolescent-specific effects, affect performance in social tasks.

Age played a role in interpretation ratings, too. It is often difficult to partial out developmental effects from age-related changes in task saliency, especially with visual stimuli (i.e., rejection from older looking peers in the picture may be confounding ratings in younger age groups). However, recent studies suggest that development may be a moderating factor in several biases relevant to emotional and mood disorders (Gamble and Rapee, 2012; Cresswell et al., 2010; Nolen-Hoeksema et al., 1986, Nolen-Hoeksema et al., 1992). It may be that adolescence represents a sensitive period for changes in risk-correlates for social anxiety: indeed, over adolescence multiple networks underpinning important functions, such as social cue interpretation and attentional control, undergo protracted maturation. Within the framework of typical developmental timelines, we can start to explore the dynamics of development over this period of plasticity and risk. This approach has the potential to reveal how normative social developmental processes may accentuate preexisting individual differences, and in turn “push” some adolescents towards the more extreme ends (Haller et al., 2013). It would be particularly interesting to examine whether there is an increase of negative interpretations at the transition to adolescence and whether mid-adolescence represents a peak in negative interpretations of ambiguous peer-related social material, compared to early and late adolescence.

### 4.1 Strengths and limitations

This study has several strengths. Firstly, it is important to assess whether mechanisms extrapolated from highly controlled stimuli (such as individual facial displays, dot-probe paradigms) still hold significance when settings are increasingly naturalistic. Using more naturalistic paradigms depicting subtle cues that could signal potential social threat (e.g. gestures, body posture, situational cues) may be a step towards an assessment of cognitive biases that reflect the demands of everyday life more closely. Ambiguity is pertinent to anxious responses in everyday life and individuals are also rarely just passive observers of social information; we orient attention to cues with the aim to discern and interpret underlying mental states that are relevant for our interactions. Hence, the integration of research on attentional allocation and interpretation biases is crucial in order to better understand the mechanisms and conditions under which attentional deployment drives appraisals and vice versa.

There are several limitations that need to be considered when interpreting the results of this study. Firstly, we did not measure depressive symptoms. The lack of an interaction between social anxiety and attention allocation may be attributable to a possible presence of depressive symptoms. Depressive symptoms frequently co-occur with social anxiety. There is some evidence to suggest that co-occurring depressive symptoms may ‘cancel out’ attentional biases linked to anxiety. A study by Taghavi et al. (1999) revealed that, while anxious adolescents, relative to controls, selectively allocated attention toward threat stimuli, adolescents with both types of symptoms did not show any attentional bias towards either threat- or depression-related material relative to participants in the control group .

Secondly, as the stimuli represented natural social scenes, we did not control for the distance between the fixation presented in the center of the scene to the focal social objects. This may have contributed to ‘washing out’ differences in attentional deployment. However, it is likely that the starting point within the scene affects initial saccades, but differences in overall fixation time are unlikely to be affected.

Thirdly, developmental conclusions need to be interpreted with caution, given that our sample did not include early adolescence, and lacked additional cross-sectional age groups. Alternatively, as pubertal status may be particularly important as an index of social-emotion processing and social anxiety, future research could also focus on pubertal status within a narrow age band.

Fourthly, it will be important for future research to assess whether these findings are generalizable to male adolescents. With puberty affecting the way adolescents interact with their peers, it may be important to select scenes in a manner that allows for the investigation of interactions between participant sex and sex of the peers displayed.

Lastly, the current study did not allow the testing of causal hypothesis regarding attention-interpretation interactions. Future studies should address this by systematically manipulating attentional focus and task demands to understand how these factors affect appraisals.

## 5 Conclusion

The dynamic, interpersonal aspect of social cognition remains under-studied. This study marks a step towards understanding the links between attentional deployment, individual differences in social anxiety and interpretations in more naturalistically portrayed social encounters. The results revealed that both attention and social anxiety independently predicted positive and negative interpretation ratings across social scenes in mid-to-late adolescent females.

## Conflict of interest

None.

## References

[bib0005] Allport A., Posner M.I. (1989). Visual attention. Foundations of Cognitive Science.

[bib0010] Amin N., Foa E.B., Coles M.E. (1998). Negative interpretation bias in social phobia. Behav. Res. Ther..

[bib0015] Amir N., Elias J., Klumpp H., Przeworski A. (2003). Attentional bias to threat in social phobia: facilitated processing of threat or difficulty disengaging attention from threat?. Behav. Res. Ther..

[bib0020] Bantin T., Stevens S., Gerlach A.L., Hermann C. (2016). What does the facial dot-probe task tell us about attentional processes in social anxiety? A systematic review. J. Behav. Ther. Exp. Psychiatry.

[bib0025] Bar-Haim Y. (2010). Research review: attention bias modification (ABM): a novel treatment for anxiety disorders. J. Child Psychol. Psychiatry.

[bib0030] Bartón, K. (2015, February 25). MuMIn: Multi-Model Inference. Retrieved from http://CRAN.R-project.org/package=MuMIn.

[bib0035] Bates D., Maechler M., Bolker B., Walker S. (2015). Fitting linear mixed-effects models using lme4. J. Stat. Softw..

[bib0040] Brainard D.H. (1997). The psychophysics toolbox. Spatial Vision.

[bib0045] Burnham K.P., Anderson D.R., Burnham K.P., Anderson D.R. (2002). Model Selection and Multi-model Inference: A Practical Information-theoretic Approach.

[bib0050] Clark D.M., Wells A. (1995). A cognitive model of social phobia. Social Phobia: Diagn. Assess. Treat..

[bib0055] Creswell C., Murray L., Cooper P. (2014). Interpretation and expectation in childhood anxiety disorders: age effects and social specificity. J. Abnorm. Child Psychol..

[bib0060] Dudeney J., Sharpe L., Hunt C. (2015). Attentional bias towards threatening stimuli in children with anxiety: a meta-analysis. Clin. Psychol. Rev..

[bib0065] Everaert J., Duyck W., Koster E.H. (2014). Attention, interpretation, and memory biases in subclinical depression: a proof-of-principle test of the combined cognitive biases hypothesis. Emotion.

[bib0070] Gamble A.L., Rapee R.M. (2009). The time-course of attentional bias in anxious children and adolescents. J. Anxiety Disord..

[bib0075] Giannini M., Loscalzo Y. (2016). Social anxiety and adolescence: interpretation bias in an Italian Sample. Scand. J. Psychol..

[bib0080] Haller S.P., Kadosh K.C., Lau J.Y. (2013). A developmental angle to understanding the mechanisms of biased cognitions in social anxiety. Front. Hum. Neurosci..

[bib0085] Haller S.P., Raeder S.M., Scerif G., Kadosh K.C., Lau J.Y. (2016). Measuring online interpretations and attributions of social situations: links with adolescent social anxiety. J. Behav. Ther. Exp. Psychiatry.

[bib0090] Hirsch C.R., Clark D.M., Mathews A. (2006). Imagery and interpretations in social phobia: support for the combined cognitive biases hypothesis. Behav. Ther..

[bib0095] In-Albon T., Kossowsky J., Schneider S. (2010). Vigilance and avoidance of threat in the eye movements of children with separation anxiety disorder. J. Abnormal Child Psychol..

[bib0100] Kessler R.C., Berglund P., Demler O., Jin R., Merikangas K.R., Walters E.E. (2005). Lifetime prevalence and age-of-onset distributions of DSM-IV disorders in the National Comorbidity Survey Replication. Arch. Gen. Psychiatry.

[bib0105] Kilford E.J., Garrett E., Blakemore S.J. (2016). The development of social cognition in adolescence: an integrated perspective. Neurosci. Biobehav. Rev..

[bib0110] La Greca A.M., Lopez N. (1998). Social anxiety among adolescents: linkages with peer relations and friendships. J. Abnorm. Child Psychol..

[bib0115] Miers A.C., Blöte A.W., Bögels S.M., Westenberg P.M. (2008). Interpretation bias and social anxiety in adolescents. J. Anxiety Disord..

[bib0120] Miers A.C., Blöte A.W., Westenberg P.M. (2011). Negative social cognitions in socially anxious youth: distorted reality or a kernel of truth?. J. Child Fam. Stud..

[bib0125] Miers A.C., Blote A.W., Heyne D.A., Westenberg P.M. (2014). Develop- mental pathways of social avoidance across adolescence: the role of social anxiety and negative cognition. J. Anxiety Disord..

[bib0130] Muris P., Field A.P. (2008). Distorted cognition and pathological anxiety in children and adolescents. Cognit. Emot..

[bib0135] Musa C.Z., Lépine J.P. (2000). Cognitive aspects of social phobia: a review of theories and experimental research. Eur. Psychiatry.

[bib0140] Nelson E.E., Jarcho J.M., Guyer A.E. (2016). Social re-orientation and brain development: an expanded and updated view. Dev. Cognit. Neurosci..

[bib0145] Nolen-Hoeksema S., Girgus J.S., Seligman M.E. (1986). Learned helplessness in children: a longitudinal study of depression, achievement, and explanatory style. J. Pers. Soc. Psychol..

[bib0150] Nolen-Hoeksema S., Girgus J.S., Seligman M.E. (1992). Predictors and con- sequences of childhood depressive symptoms: a 5-year longitudinal study. J. Abnorm. Psychol..

[bib0155] Norman D.A., Shallice T., Davidson R., Schwartz G., Shapiro D. (1986). Attention to action: willed and automatic control of behavior.

[bib0160] Nyström M., Holmqvist K. (2010). An adaptive algorithm for fixation, saccade, and glissade detection in eyetracking data. Behav. Res. Methods.

[bib0165] Petersen A.C., Crockett L., Richards M., Boxer A. (1988). A self-report measure of pubertal status: reliability, validity, and initial norms. J. Youth Adolesc..

[bib0170] Price R.B., Siegle G.J., Silk J.S., Ladouceur C., McFarland A., Dahl R.E., Ryan N.D. (2013). Sustained neural alterations in anxious youth performing an attentional bias task: a pupilometry study. Depress. Anxiety.

[bib0175] Puliafico A.C., Kendall P.C. (2006). Threat-related attentional bias in anxious youth: a review. Clin. Child Fam. Psychol. Rev..

[bib0180] Seefeldt W.L., Kramer M., Tuschen-Caffier B., Heinrichs N. (2014). Hypervigilance and avoidance in visual attention in children with social phobia. J. Behav. Ther. Exp. Psychiatry.

[bib0185] Shechner T., Jarcho J.M., Britton J.C., Leibenluft E., Pine D.S., Nelson E.E. (2013). Attention bias of anxious youth during extended exposure of emotional face pairs: an eye-tracking study. Depress. Anxiety.

[bib0190] Shechner T., Jarcho J.M., Wong S., Leibenluft E., Pine D.S., Nelson E.E. (2015). Threats, rewards, and attention deployment in anxious youth and adults: an eye tracking study. Biol. Psychol..

[bib0195] Silk J.S., Stroud L.R., Siegle G.J., Dahl R.E., Lee K.H., Nelson E.E. (2012). Peer acceptance and rejection through the eyes of youth: pupillary, eyetracking and ecological data from the chatroom interact task. Soc. Cogn. Affect. Neurosci..

[bib0200] Steinberg L., Silverberg S.B. (1986). The vicissitudes of autonomy in early adolescence. Child Dev..

[bib0205] Storch E.A., Masia-Warner C., Dent H.C., Roberti J.W., Fisher P.H. (2004). Psychometric evaluation of the Social anxiety scale for adolescents and the social phobia and anxiety inventory for children: construct validity and normative data. J. Anxiety Disord..

[bib0210] Taghavi M.R., Neshat-Doost H.T., Moradi A.R., Yule W., Dalgleish T. (1999). Biases in visual attention in children and adolescents with clinical anxiety and mixed anxiety-depression. J. Abnorm. Child Psychol..

[bib0215] Tulbure B.T., Szentagotai A., Dobrean A., David D. (2012). Evidence based clinical assessment of child and adolescent social phobia: a critical review of rating scales. Child Psychiatry Hum. Dev..

[bib0220] Vassilopoulos S.P., Banerjee R. (2012). Social anxiety and content specificity of interpretation and judgemental bias in children. Infant Child Dev..

[bib0225] Westenberg M.P., Drewes M.J., Goedhart A.W., Siebelink B.M., Treffers P.D. (2004). A developmental analysis of self-reported fears in late childhood through mid-adolescence: social-evaluative fears on the rise?. J. Child Psychol. Psychiatry.

[bib0230] White L.K., Suway J.G., Pine D.S., Bar-Haim Y., Fox N.A. (2011). Cascading effects: the influence of attention bias to threat on the interpretation of ambiguous information. Behav. Res. Ther..

[bib0235] Wong Q.J., Rapee R.M. (2016). The aetiology and maintenance of social anxiety disorder: a synthesis of complimentary theoretical models and formulation of a new integrated model. J. Affect. Disord..

