# Supplementary material for: Attention allocation and social worries predict interpretations of peer-related social cues in adolescents
Source: Dev Cogn Neurosci. 2017 Mar 18;25:105–12. doi: 10.1016/j.dcn.2017.03.004 (PMC5485637; doi:10.1016/j.dcn.2017.03.004)
Supplement: Supplementary file 1 [file mmc1.docx]

*Supplementary materials*

*Pupil data*

Pupil diameter data was smoothed with a 3-sample (100 ms) median filter. Pupil diameter was baseline corrected with respect to the mean of the 200 ms prior to scene onset. Invalid data for pupil diameter was linearly interpolated.

*Results*

Data points represent means across 5000 ms viewing period for each scene. We controlled for number of fixations and scene luminance. As for the previous models, coefficient estimates for the two-way interaction between interpretation valence and social anxiety were significant. Additionally, a three-way interaction emerged between interpretation valence, social anxiety, and pupil size. The three-way interaction showed that for youths with high levels of social worries pupil size was not predictive of positive and negative interpretation ratings. For youths with low levels of social worries, pupil size was linked to negative and positive interpretation ratings with increased pupil size related to increased negative ratings and decreased positive ratings. Results are in line with Price and colleagues (2013) suggesting that high anxious individuals display an inflexible pattern of pupillary responding.

| Fixed effect | Estimate | 95% CI |  | *p*-value |
| --- | --- | --- | --- | --- |
| **(Intercept)** | **52.16** | **[47.25,** | **57.08]** | **<0.001** |
| Number of fixations | -0.28 | [-0.61, | 0.06] | 0.108 |
| Interpretation valence | -1.87 | [-4.65, | 0.96] | 0.194 |
| Social anxiety | 1.25 | [-0.61, | 3.11] | 0.193 |
| Scene luminance | -0.93 | [-2.08, | 0.22] | 0.115 |
| **Interpretation valence*Social anxiety** | **-2.22** | **[-4.25,** | **-0.15]** | **0.036** |
| Perspective | -0.52 | [-3.46, | 2.44] | 0.731 |
| Perspective*Interpretation valence | 3.36 | [-0.58, | 7.30] | 0.095 |
| Pupil size | 0.27 | [-1.10, | 1.65] | 0.713 |
| Interpretation valence*Pupil size | -0.68 | [-2.79, | 1.34] | 0.531 |
| Pupil size*Social anxiety | -0.84 | [-2.67, | 0.82] | 0.349 |
| **Interpretation valence*Pupil size*Social anxiety** | **2.23** | **[0.12,** | **4.34]** | **0.038** |
| Perspective*Social anxiety | -0.31 | [-2.54, | 1.92] | 0.789 |
| Perspective*Interpretation valence*Social anxiety | 1.68 | [-2.24, | 5.65] | 0.403 |
| Perspective*Pupil size | 0.51 | [-1.68, | 2.61] | 0.654 |
| Perspective*Interpretation valence*Pupil size | -2.08 | [-6.07, | 1.84] | 0.303 |
| Perspective*Pupil size*Social anxiety | -1.15 | [-3.29, | 0.99] | 0.294 |
| Perspective*Interpretation valence*Pupil size*SA | 1.15 | [-3.07, | 5.37] | 0.594 |


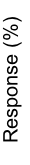

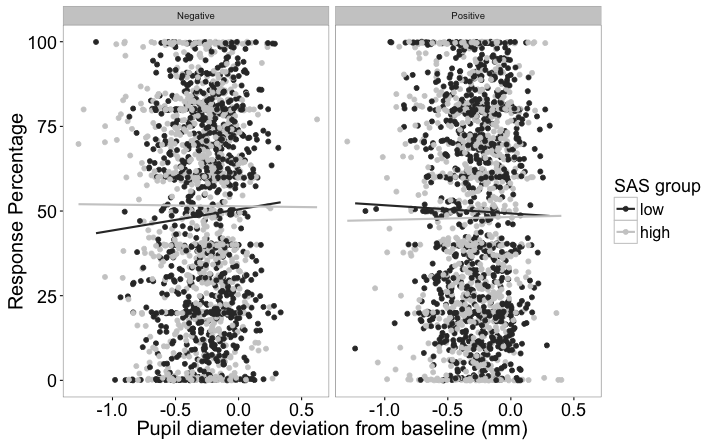


Social anxiety
